# Statistics in the Operating Room: A Cardiovascular Surgeon’s Guide to Numbers That Matter

**DOI:** 10.7759/cureus.54151

**Published:** 2024-02-13

**Authors:** Vishal V Bhende, Tanishq S Sharma, Mathangi Krishnakumar, Jaishree D Ganjiwale, Anikode Subramanian Ramaswamy, Kanchan Bilgi, Sohilkhan R Pathan

**Affiliations:** 1 Pediatric Cardiac Surgery, Bhanubhai and Madhuben Patel Cardiac Centre, Shree Krishna Hospital, Bhaikaka University, Karamsad, IND; 2 Community Medicine, SAL Institute of Medical Sciences, Ahmedabad, IND; 3 Anesthesiology, St John's Medical College Hospital, Bengaluru, IND; 4 Biostatistics and Epidemiology, Pramukh Swami Medical College, Bhaikaka University, Karamsad, IND; 5 Pathology, PES Institute of Medical Sciences and Research, Kuppam, IND; 6 Neuroanesthesiology, People Tree Hospitals, Bengaluru, IND; 7 Clinical Research, Bhanubhai and Madhuben Patel Cardiac Centre, Shree Krishna Hospital, Bhaikaka University, Karamsad, IND

**Keywords:** data, survival analysis, hypothesis testing, pediatric cardiac surgery, statistics

## Abstract

Pediatric cardiac surgery demands meticulous technique, but optimal outcomes hinge on translating data into actionable insights. This editorial bridges the gap between scalpel and statistical jargon, empowering surgeons to decipher common tests. Descriptive statistics paint portraits of patient cohorts, while hypothesis testing discerns real differences from chance. Regression analysis unveils hidden relationships, predicting outcomes based on complex interplays of variables. Survival analysis tracks the delicate dance of time and survival, informing therapeutic strategies. By embracing statistical fluency, surgeons become architects of personalized care, tailoring interventions to mitigate risks and maximize the precious gift of a beating heart.

## Editorial

The sterile world of pediatric cardiothoracic surgery thrives on precision, where every stitch and maneuver holds the fate of a tiny heart in the balance. Yet, amidst the scalpel and suture, another language takes hold, the language of statistics. These seemingly arcane calculations, often relegated to the realm of biostatisticians, hold the key to unlocking insights that can revolutionize surgical practice, improve outcomes, and ultimately, save lives [1]. For many surgeons, statistics can seem like a daunting labyrinth. But just as a surgeon meticulously masters anatomical intricacies, so too can they navigate the statistical terrain with confidence. This editorial aims to demystify some of the most commonly encountered statistical tests in pediatric cardiothoracic surgery, equipping surgeons with the tools to interpret and utilize these valuable resources.

The art of interpretation: various types

(A) Descriptive Statistics: Painting a Portrait of the Data

Before diving into hypothesis testing, a basic understanding of the data is crucial. Descriptive metrics such as the mean (M1), median (M2), mode (M3), standard deviation (SD), and inter-quartile range (IQR) are employed for descriptive statistical analysis, which provides a snapshot of the quantitative data, summarizing its central tendency and spread. For instance, analyzing the mean post-operative length of stay in a cohort of patients undergoing atrial septal defect (ASD) closure paints a picture of how long, on average, these patients require hospitalization. Mean should be supplemented with SD and median with IQR to depict the average along with the spread. For categorical data, simple frequency (%)is used to describe.

(B) Hypothesis Testing: Validating Our Assumptions

Clinical research often hinges on testing specific hypotheses about the relationship between two variables. For example, a surgeon might hypothesize that using a specific surgical technique for aortic stenosis repair leads to lower rates of re-intervention. Statistical tests like the independent sample t-test or analysis of variance (ANOVA) help determine whether observed differences between groups are due to chance or reflect a true underlying relationship. Independent sample t-test compares means of two groups for continuous variables (e.g., height, weight, test scores), and ANOVA is for when there are more than two groups. Post hoc tests are used to locate the difference precisely after ANOVA; you find a significant difference in ANOVA. For examining the association between categorical variables (e.g., gender, blood type, treatment outcomes) a Chi-square test is used.

(C) Correlation and Regression: Unveiling Hidden Relationships

While hypothesis testing focuses on determining associations and/or comparisons between groups, correlation analysis explores the strength and direction of association between two continuous variables. For instance, a study might investigate the relationship between pre-operative weight and post-operative recovery time in patients undergoing congenital heart surgery. A regression analysis will depict this relation through an equation quantifying the precise nature of the relationship to allow for the prediction of one variable based on the other if needed. A multivariable linear regression explores relationships between multiple independent variables (predictors) and a continuous dependent variable (outcome). A binary logistic regression predicts the probability of a binary outcome (Yes/No, True/False, Success/Failure).

(D) Survival Analysis: When Time Matters

In pediatric cardiac surgery, the ultimate measure of success is often patient survival. Survival analysis tools like the Kaplan-Meier curve and log-rank test help researchers compare survival rates between groups and identify factors that influence long-term outcomes. For example, a surgeon might utilize survival analysis to compare the long-term survival rates of patients undergoing two different Fontan procedures for hypoplastic left heart syndrome (HLHS).

(E) Risk Stratification: Predicting the Future

Every patient presents a unique set of risk factors influencing their surgical outcome. Risk stratification models, powered by statistical tools like multivariate logistic regression, combine various patient characteristics to predict their individual risk of complications or mortality for different characteristics. This allows surgeons to tailor surgical strategies and optimize care for each patient, ultimately improving outcomes for high-risk individuals. The Cox regression analysis method goes beyond simply knowing about the odds of a child eventually experiencing a complication. It helps understand how factors like pre-operative risk scores, surgical techniques, or genetic profiles influence the speed of arrival for those events. Table 1 depicts the statistical tests discussed in the paper.

**Table 1:** Summary of some statistical tests for easy understanding

Sr. No.	Category	Method	Description	Example use case
1	Descriptive statistics	Mean, median, mode, standard deviation, frequency (%) tables	Summarize and describe a set of data; understand central tendency and spread	Characterizing pre-operative patient characteristics, simply presenting groups of patients based on demographics
2	Hypothesis testing	t-tests (paired/independent sample), ANOVA (one-way/two-way/repeated measures), Chi-square test followed by post hoc analysis	Assess whether there is a statistically significant disparity between groups or if a variable is linked to a specific outcome	Comparing surgical outcomes between minimally invasive and open approaches, evaluating the impact of a new surgical technique on complication rates
3	Regression analysis	Linear regression, logistic regression, cox proportional hazards model	Assess the relationship between one or more independent variables and a continuous or categorical outcome variable	Identifying risk factors for post-operative complications, predicting long-term survival after surgery
4	Survival analysis	Kaplan-Meier curves, log-rank test	Analyze time-to-event data, such as time to death or time to recurrence	Comparing survival rates after different cardiac surgery procedures, evaluating the effectiveness of a new treatment for improving long-term survival

Numbers with a story: statistics in action

Let us illustrate these concepts with real-world examples from pediatric cardiothoracic surgery:

(A) Descriptive Statistics

Example 1: A study by Passos et al. investigated the outcomes of minimally invasive mitral valve surgery in a diverse cohort of valve pathologies. The analysis examined data from 278 patients undergoing mitral valve surgery. The main objective was to understand various valve pathologies. Descriptive statistics revealed that the most common procedures were resection (29.5%), neo-chordae implantation (23.7%), and a combination of both (6.1%). All preoperative variables, including bypass time (164 ± 47 minutes) and cross-clamp time (106 ± 36 minutes), were similar across the group. Degenerative valve disease was the predominant pathology, with Barlow (20.5%), bi-leaflet (20.5%), and double segment (32.4%) being the most prevalent types [2].

Example 2: A study focused on the prevalence and risk factors for post-operative arrhythmias after pediatric heart surgery. Post-operative arrhythmias posed a significant challenge, affecting 17.1% (140) of the 821 patients. Junctional ectopic tachycardia (JET) dominated, occurring in 51.4% of cases, followed by atrioventricular block (27.1%) and supraventricular tachycardia (10%). Notably, 79.3% of arrhythmias arose within the first 24 hours after surgery [3].

(B) Hypothesis Testing

Example 1: A study by Van de Heyde et al. (2023) compared two surgical approaches for treating atrial ASDs in children: standard open repair and mini-thoracotomy approach. Using an independent sample t-test, they found a statistically significant difference in length of hospital stay, with shorter stay in the mini-thoracotomy group (p < 0.05). This suggests potential advantages of the minimally invasive approach [4].

Example 2: Another study by Lin et al. (2020) investigated the association between pre-operative levels of brain natriuretic peptide (BNP) and post-operative outcomes in individuals undergoing Fontan surgery. A Chi-square test revealed a significant association between elevated BNP levels and the occurrence of low cardiac output syndrome (p < 0.05). This suggests BNP as a potential predictor of complications in this high-risk group [5].

(C) Regression Analysis

Example 1: A study by Mavroudis et al. (2023) in pediatric patients undergoing ventricular septal defect (VSD) repair aimed to determine the anticipated duration of post-operative intensive care unit (ICU) stay and mechanical ventilation. A multivariable linear regression identified several significant predictors of ICU stay, including genetic syndromes, extended bypass duration, and reduced body weight during surgery. This model could help optimize resource allocation and patient expectations [6].

Example 2: In a recent study, Zheng et al. investigated the efficacy of three established heart transplant risk scores, Mortality Prediction After Cardiac Transplantation (IMPACT), the United Network for Organ Sharing (UNOS), and risk stratification scores (RSSs), in predicting one-year mortality using binary logistic regression. All participants received scores on each system. Analysis revealed statistically significant odds ratios (OR) for mortality prediction by all three scores. IMPACT OR 1.25 (95% CI 1.15-1.36, p < 0.001), UNOS (OR 1.68, 95% CI 1.37-2.07, p < 0.001), and RSS (OR 1.61, 95% CI 1.30-2.00, p < 0.001) were determined in the analysis. These findings suggest the potential of these scoring systems to aid in patient selection and risk stratification for heart transplantation [7].

(D) Survival Analysis

Example 1: In a retrospective study investigating long-term outcomes after the Glenn procedure alone in single-ventricle patients, Miyake et al. employed Kaplan-Meier curves to reveal a 40.3% 40-year survival rate. Notably, Individuals demonstrating dominant left ventricular morphology have a potential correlation with enhanced overall survival when compared with their counterparts exhibiting dominant right ventricular morphology [8].

Example 2: This extensive study of 343 infants with HLHS and related complex congenital heart defects employed survival analysis and Cox regression to paint a detailed picture of their long-term outcomes. Using Kaplan-Meier curves, they revealed encouraging survival rates, reaching 84.4% at one week and 32.6% at 15 years. Notably, Cox regression identified prematurity, low birth weight, and female sex as significant risk factors for mortality. This comprehensive analysis highlights the improved prognosis for HLHS in recent years, owing to progress in healthcare and advancements in surgical methodologies [9]. 

Choosing the right statistical test: a stepwise approach

Step 1

Define your research question and hypothesis: (a) What are you trying to understand or prove? (b) Is your focus on describing data, testing hypotheses, or predicting outcomes?

Step 2

Identify your data type: (a) Is it nominal (categories), ordinal (ranked categories), interval (ordered numbers), or ratio (true zero and proportional differences)? (b) Are your variables continuous or discrete?

Step 3

Consider the number of variables involved: (a) Are you analyzing one dependent variable (outcome) with several independent variables (predictors)? (b) Or are you exploring relationships between multiple variables without a specific dependent variable?

Step 4

Choose the appropriate statistical test based on your answers to the above. 

**Figure 1:**
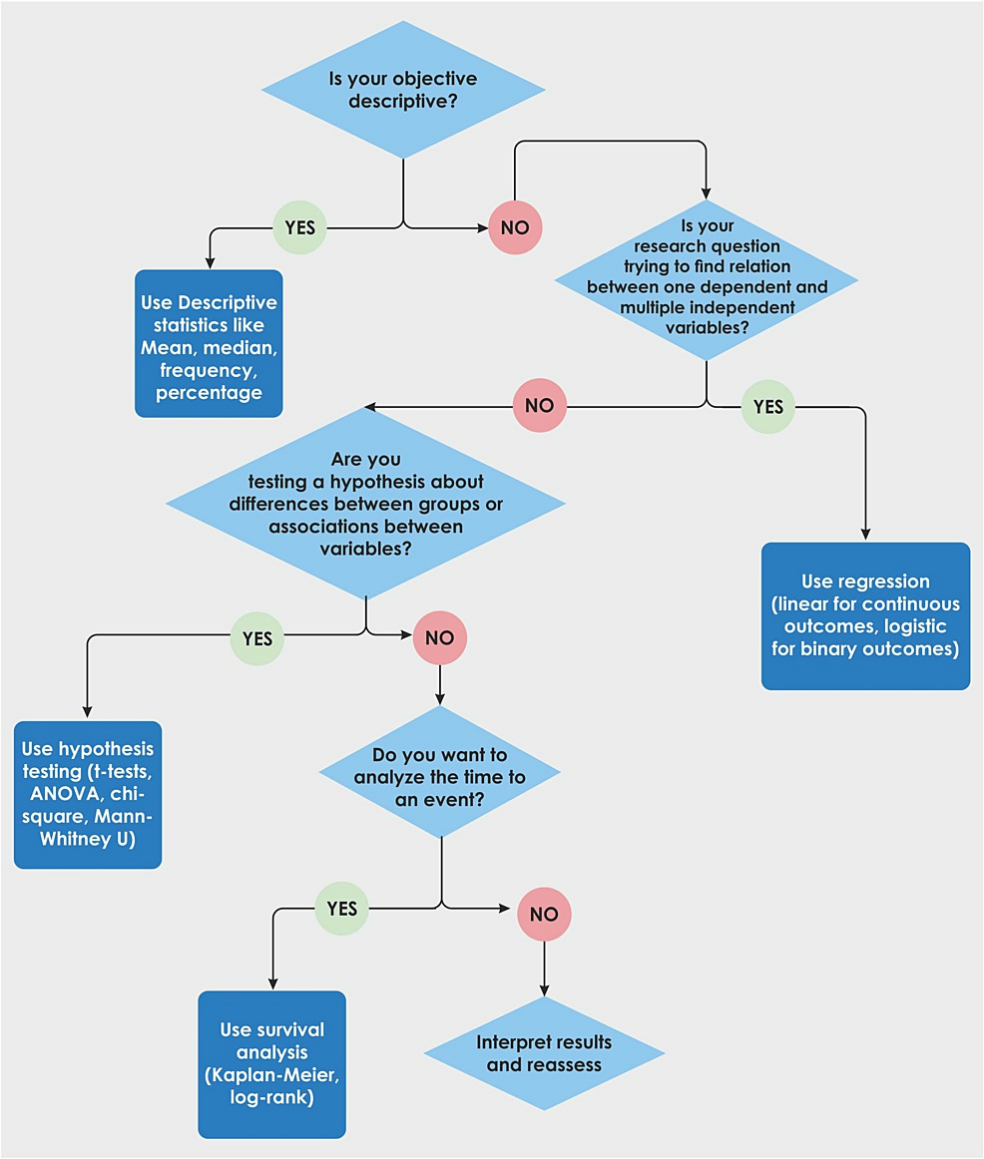
Stepwise approach to statistical analysis Image credits: Dr. Mathangi Krishnakumar

Figure 1 depicts a stepwise approach to a few common research questions.

While we have covered numerous examples and statistical tests, the landscape extends far beyond what we've discussed. Your specific research objective will determine the most appropriate approach, whether it is any situation we already mentioned here or something different. It may be delving into non-parametric tests or a sensitivity analysis or correlation analysis or conducting a method comparison study or exploring factors through exploratory factor analysis or confirming existing findings through confirmatory factor analysis. Ultimately, the most fitting analysis will depend on your research context, specific questions, and chosen study design. It is important to have a proper understanding of statistics. One may need to consult an expert for this all through, starting from formulating a research study till interpreting the results.

Conclusion

Statistical tests alone are not magic bullets. The findings of the tests should be interpreted in light of the context and potential biases in the study. Just as a surgeon would not rely solely on a single X-ray to diagnose a complex condition, so too should statistical results be considered alongside clinical expertise and a holistic understanding of the data. If this requires collaboration with people with a better understanding of statistics, you should go ahead and make your team!
